# St. Luke’s Hospital

**Published:** 1877-12

**Authors:** 


### Clinical Reports.

### ST. LUKE'S HOSPITAL.

Service of Jno. E. Owens, M. D.,

[Prof. Surgery, Woman's Med. College ; Lecturer on Surg., Rush Med. College, etc.]

### *Lacerated Wound of Fore-arm; Tetanus; Treatment by Calabar Bean; Stretching of Brachial Plexus; Death.*

Reports of cases of traumatic tetanus, *successfully* treated by one method or another, are so numerous in current medical literature, that to judge from such reports alone, one must conclude that the treatment of the disease in question is tolerably satisfactory. I therefore publish this unsuccessful case, since it is only by a study of the successful and unsuccessful that a proper estimate can be placed upon this or that mode of management.

Aug. 29, 1877, a little boy, aged six and a half years, was injured by a passing freight train. I saw the case a short time after the injury, with Dr. G. M. Chamberlin, who was already in attendance. The integument, with little or no loss of substance, had been torn from the front of the right forearm. The laceration extended to the palm of the hand, and involved, to some extent, the base of the thumb. The temporary dressings were saturated with blood, the arm and hand being warm. We placed in apposition the margins of the lacerated integuments, except for two or three inches at the wrist, and introduced the requisite number of sutures.

The following day, Aug. 30, the patient was admitted to the hospital. The arm was dressed with a warm solution of carbolic acid. On the third day, however, the skin showed signs of mortification, which extended, until all of the lacerated skin and that of the fingers and the back of the hand became involved. The integument of the fingers and dorsum of the hand furnished no evidence of injury when first seen.

The patient had several attacks of "night terrors" in the hospital; but his father reported that he was subject to these previous to the injury.

The fifth day, the resident began to remove the dead integument, small portions still remaining on the palm of the hand and on the back of the wrist on the tenth day. The case, however, in other respects progressed very favorably. The patient ate and slept well, except the occasional disturbance above referred to, until the morning of Sept. 8th, when Dr. Brown, the resident, reported that the patient cried out at certain intervals from pain in the arm, induced by spasm. Upon attempting to place the thermometer under the tongue, it was found that the jaws were so rigid that the instrument could not be safely used. Jaws could be opened a quarter of an inch.

TEMPERATURE AND PULSE RECORD.

| | Temperature. | | Pulse. | |
|---|---|---|---|---|
| | A. M. | P. M. | A. M. | P. M. |
| Aug. 31, | - | 103° | - | 136 |

| | | Temperature. | | | | Pulse. | | |
|---|---|---|---|---|---|---|---|---|
| | | A. M. | | P. M. | | A. M. | | P. M. |
| Sept. | 1, | $100\frac{4}{5}$ | - | $101\frac{3}{5}$ | - | 112 | - | 128 |
| " | 2, | $100\frac{1}{5}$ | - | $100\frac{3}{5}$ | - | 118 | - | 120 |
| " | 3, | 100 | - | $100\frac{1}{5}$ | - | 118 | - | 130 |
| " | 4, | $99\frac{4}{5}$ | - | 101 | - | 122 | - | 140 |
| " | 5, | $100\frac{1}{3}$ | - | $101\frac{1}{3}$ | - | 118 | - | 130 |
| " | 6, | $100\frac{1}{3}$ | - | 101 | - | 124 | - | 136 |
| " | 7, | $100\frac{2}{3}$ | - | $101\frac{1}{3}$ | - | 122 | - | 136 |
| " | 8, | | - | | - | 118 | - | |

The resident had prescribed the following :

℞ Potas. bromid. - - - ʒ v.

Morphia sulph. - - - gr. $\frac{1}{4}$.

Aquæ - - - - - ℥ ii. M.

S. Teaspoonful every 2 hrs.

Upon reaching the hospital, I found the patient asleep. In the afternoon the jaws had relaxed somewhat. The arm was enveloped in warm poultices.

Sept. 9.—Patient could open mouth three-quarters of an inch, and was free from pain, except when the arm was disturbed by spasm of its muscles. Treatment continued. At noon it was reported that the patient had slept the greater part of the morning, but the spasms of the arm had become more frequent, the interval ranging, for a time, from three to five minutes. The muscles of the neck were rigid and the head bent back. The patient complained of his throat, and refused to swallow his medicine.

At 2:30 P. M. there supervened a violent spasm of the muscles of the jaws, neck and trunk, opisthotonos being so great that the vertex was buried in the pillow. In a half hour the jaws could be separated three quarters of an inch, the patient was perspiring profusely, and the face flushed. The opisthotonos and rigidity of the affected muscles remained. Three seizures occurred within ten minutes.

3:45 P. M. Ordered subcutaneous injection of morphia, $\frac{1}{6}$ gr.

At 3:57 P. M., whilst sleeping, he had another opisthotonic seizure, of momentary duration, which awoke him. He gave a cry of distress and dropped off to sleep again.

6 P. M. Opisthotonos pronounced, paroxysms frequent and severe; rigidity constant.

6:45 P. M. Morphia and bromide of potash discontinued; Squibb's alcoholic extract of Calabar bean, gr. $\frac{1}{11}$ substituted.

7 P. M. Calabar bean, gr. $\frac{1}{11}$.

7:15 P.M. " " "

7:30 P.M. " " " by hypodermic injection.

The frequency of the paroxysms became notably diminished, recurring at intervals, varying from five to fifteen. This improvement was temporary, for in an hour the intervals ranged from three to five minutes.

8:45 P.M. Calabar bean, gr. $\frac{1}{11}$, by hypodermic injection.

10:30 P.M. " " " " " .

Sept. 10th.—12:30 A.M. Calabar bean, gr. $\frac{1}{11}$; pulse 120; respirations 26; result of medication since 8:45 P. M., Sept. 9th, negative.

1:15 A.M., Calabar bean, gr. $\frac{1}{11}$.

1:30 A.M. " "

2:00 A.M. " "

2:15 A.M. " "

2:30 A.M. " "

3:15 A.M. " "

3:30 A.M. " "

4:00 A.M. " "

4:15 A.M. " "

4:45 A.M. " "

A new solution of Calabar bean, prepared at 5 A.M., each drachm of which contained gr. $\frac{1}{7}$.

5:00 A.M., Calabar bean, gr. $\frac{1}{7}$th.

5:45 A.M., " " "

6:30 A.M., " " gr. $\frac{2}{7}$th.

7:05 A.M., " " gr. $\frac{1}{11}$th, hypodermic injection.

8:15 A.M., " " " $\frac{2}{7}$th

9:20 A.M., " " " paroxysms very short and less severe.

9:45 A.M., " " gr. $\frac{1}{7}$th.

11:15 A.M., " " " $\frac{2}{7}$th.

12:15 P.M., " " " $\frac{2}{7}$th; paroxysms recurred at intervals, varying from three to five minutes, but they were very light, amounting only to a momentary contraction of the muscles.

1:30 P.M. Calabar bean, gr. $\frac{2}{7}$th; pulse 144; respirations 28, and superficial.

2:30 P.M. Calabar bean, gr. $\frac{2}{7}$th. As it was apparent that the case was rapidly approaching a fatal termination, under medical treatment, at 4:30 P.M., assisted by Drs. S. J. Jones, Chamberlin and Richy, I cut down upon and stretched the brachial plexus.

An incision, two inches in length, and terminating below at the clavicle, was made, parallel to the anterior margin of the trapezius muscle, in the space between the latter and the external jugular vein. By deeper incisions through the superficial fascia, the platysma, and some cellular tissue and fat, the plexus was exposed in the triangle formed by the posterior belly of the omohyoid, the trapezius and the levator anguli scapulæ muscles. The sheath, to all appearances normal, was slit up as far as the intervertebral foramina. The plexus having then been lifted from the sheath by means of an aneurism needle, the flexed index finger was substituted for the needle and traction exerted upon both the central and peripheral portions. The wound was then washed with carbolic lotion, and closed by interrupted suture, except at its most dependent part, in which a small tent was placed. For a half hour after the operation, the patient's condition seemed to have improved. The convulsive seizures ceased, the opisthotonos subsided, the patient resting the back of the head instead of the vertex on the pillow, but a certain degree of rigidity of the cervical and other muscles remained. The patient was able to separate the jaws three quarters of inch. This apparent improvement, due, in my opinion, to ether, soon passed away, after which the convulsive attacks were more violent than they had ever been.

7:30 P.M. Aqueous ext. Calabar bean, $\frac{2}{5}$ gr. (hypodermic).

7:45 P.M. Calabar bean, (hypodermic) gr. $\frac{1}{2}$.

8 P.M. The paroxysms were so severe, that I asked the resident to give the patient a few inhalations of chloroform.

Already somewhat under the influence of Calabar bean, the patient ceased to breathe after he had inhaled a very small quantity of chloroform. By drawing the tongue forward and continuing artificial respiration for a few minutes, respiration became re-established.

8:15 P.M. Another spasm; Calabar bean, $\frac{1}{2}$ gr. (hypodermic).

9:00 P.M. Calabar bean, gr. $\frac{1}{2}$ (hypodermic).

9:30 P.M. " " " "

10:00 P.M. " " " (hypodermic); great thirst, but the patient instantly regurgitates any fluid that he attempts to swallow.

10:30 P.M. No abatement of the spasms; increased thirst.

11 P.M. Calabar bean, gr. $\frac{1}{2}$; patient insensible to the introduction of the needle of the hypodermic syringe.

11:30 P.M. Calabar bean, gr. $\frac{1}{2}$; pulse 170.

12:00, midnight. Calabar bean, gr. $\frac{1}{2}$; patient still conscious.

Sept. 11th—12:45 A.M. Calabar bean, gr. $\frac{1}{2}$; paroxysms less severe.

1:30 A.M. Calabar bean, gr. $\frac{1}{2}$; hurried respirations.

2:15 A.M. Calabar bean, gr. $\frac{1}{2}$.

3:00 A.M. Asleep; pulse 180.

4:00 A.M. Great suffering; inhaled ether more or less till 5 A.M.

6:00 A.M. Extreme thirst; inability to swallow; pulse 180; respiration 48.

7:00 A.M. Pulse 180; respiration 48; lips cyanosed; pupils strongly contracted.

7:45 A.M. Died.

TEMPERATURE AFTER DEATH.

